# Subcortical Origin of Salience Processing Deficits in Schizophrenia

**DOI:** 10.1016/j.bpsgos.2021.12.008

**Published:** 2022-01-11

**Authors:** Lena Palaniyappan

**Affiliations:** Department of Psychiatry, Schulich School of Medicine and Dentistry, Western University, London, Ontario, Canada; Department of Medical Biophysics, Western University, London, Ontario, Canada; Robarts Research Institute, Western University, London, Ontario, Canada; Lawson Health Research Institute, London, Ontario, Canada

In the current issue of *Biological Psychiatry: Global Open Science*, Kesby *et al.* (1) provide one of the most comprehensive overviews on the neural circuitry of salience and reward processing in psychosis. Based on their thought-provoking synthesis, the authors put forward an important argument: that salience and reward processing dysfunction seen in psychosis originates in the subcortical regions, with cortical abnormalities arising only later in established stages of schizophrenia. They also list several cortical areas (the orbitofrontal, ventromedial, and dorsolateral prefrontal cortices and the ventral and superior frontal and dorsal anterior cingulate cortices) as having distinct roles in representing salience, reward processing, and the precision of prediction error signals.

SEE CORRESPONDING ARTICLE ON PAGE 33

First, their interesting hypothesis of subcortico-cortical progression is built on reports of excess striatal dopamine synthesis capacity in early at-risk stages of illness, defined by the presence of subthreshold positive symptoms. In this regard, it is important to note that more recent meta-analytical evidence is less convincing of striatal hyperdopaminergia in at-risk subjects (2). In fact, the effect size of the reported presynaptic dopamine excess in clinical high-risk individuals has decreased over time since the work that Kesby *et al.* (1) cite in favor of this argument was published (3). While this can be attributed to reduced transition rates, indicating a reduction in the number of subjects with “early stage of schizophrenia” in recent cohorts, meta-analysis of variance does not support the presence of higher interindividual variation that may occur if subgroups with hyperdopaminergic profile were present (2). The genetic high-risk groups also fail to show elevated striatal dopamine synthesis capacity (2).

A recent study comprised entirely of antipsychotic drug–naïve patients with first-episode psychosis failed to reveal an excess of dopamine synthesis capacity despite the relatively limited duration of untreated psychosis (4). Nevertheless, in this study, patients with higher positive symptom burden at baseline had higher dopamine synthesis capacity. Similarly, in patients with more established illness, reduced striatal dopamine synthesis capacity is associated with more prominent negative symptoms (5). Taken together, we can expect a subtype of patients with more insidious onset with less prominent positive symptoms but more negative symptoms to have no striatal hyperdopaminergia on presentation but instead have a “cortical origin schizophrenia.” In other words, the sequence of anatomical changes describing disease progression may not be the same across all subjects. This possibility needs to be considered in future longitudinal studies that empirically test the hypothesis of subcortical origin of salience processing deficits.

Second, of the several cortical areas listed as key components of salience and prediction error processing in psychosis, there is an intriguing absence of the anterior insula. The anterior insula is seen as the seat of a cortical salience processing system, often termed the salience network (6). One of the most replicated structural observations in schizophrenia is the bilateral reduction of gray matter in the anterior insular cortex (7). Of direct relevance to their synthesis of prediction error literature, a recent meta-analysis cited by Kesby *et al.* (1) indeed highlights the striatum and insula as the two core regions tracking domain-general prediction error signals (8).

The unusual overweighting of perceptual priors seen as a crucial feature of hallucinations relates to anterior insular activity (9). Limongi *et al.* (10) originally hypothesized a role for the anterior insula in overweighting priors to compensate for aberrant precision weighting of prediction errors in psychosis and found empirical evidence from drift-diffusion modeling of Stroop task performance in patients with first-episode psychosis in support (11). Thus, aberrant striatal dopamine signaling in psychosis, via inappropriate weighting of prediction error signals (i.e., excess precision afforded to discrete perceptual events), may propagate hierarchically to the anterior insula, generating the response of overweighted priors. But this kind of cortical response is unlikely to be a late-in-the-illness effect, as experimental manipulation of dopamine levels results in immediately observable changes in how cortical salience processing regions track beliefs about the environment (12). Furthermore, overweighting of priors appear to have a role in hallucinations (13), which, if any, are less prominent in late than in earlier stages of schizophrenia (14).

The subcortical dopamine dysfunction and cortical information processing deficits are likely to be two component parts of a pathophysiological continuum. While we currently do not know which of these two appear first in the course of this illness, the full spectrum of clinical expression may necessitate some degree of concomitant disruption in both component parts.
